# Effect of Bufei Yishen Granules Combined with Electroacupuncture in Rats with Chronic Obstructive Pulmonary Disease via the Regulation of TLR-4/NF-*κ*B Signaling

**DOI:** 10.1155/2019/6708645

**Published:** 2019-05-29

**Authors:** Jindi Ma, Yange Tian, Jiansheng Li, Lanxi Zhang, Mingming Wu, Lihua Zhu, Shuai Liu

**Affiliations:** ^1^Dongzhimen Hospital, Beijing University of Chinese Medicine, Beijing 100700, China; ^2^Collaborative Innovation Center for Respiratory Disease Diagnosis and Treatment & Chinese Medicine Development of Henan Province, Henan University of Chinese Medicine, Zhengzhou, Henan 450046, China; ^3^Henan Key Laboratory of Chinese Medicine for Respiratory Disease, Henan University of Chinese Medicine, Zhengzhou, Henan 450046, China

## Abstract

**Background:**

The combined therapy of Bufei Yishen granules (BY) and electroacupuncture (EA) has shown good effects clinically in treating chronic obstructive pulmonary disease (COPD). The present study aimed to observe the effects of the BY + EA combination in a COPD rat model and dissect the potential mechanisms via Toll-like receptor (TLR) 4/nuclear factor kappa B (NF-*κ*B) signaling.

**Methods:**

The COPD rats were treated with normal saline, aminophylline, Bufei Yishen granules, electroacupuncture, or Bufei Yishen granules combined with electroacupuncture. The pulmonary function; lung tissue histology; levels of inflammatory factors; expression levels of TLR-4, inhibitor of nuclear factor kappa B (I*κ*B), and NF-*κ*B; and activation of NF-*κ*B in the lung tissues were evaluated.

**Results:**

Pulmonary function was markedly decreased in the COPD rats, and the lung tissue histology of the COPD rats showed severe pathological changes. The pulmonary function and lung tissue morphology in the treatment groups (APL, BY, EA, and BY + EA) were improved. The increased levels of the inflammatory cytokines interleukin (IL)-1*β* and IL-6 indicated a chronic inflammatory state in the COPD rats. In the BY, EA, and BY + EA groups, the levels of IL-1*β* and IL-6 were decreased, especially in the BY + EA group. In addition, the mRNA and protein expression levels of TLR-4, I*κ*B, and NF-*κ*B were obviously downregulated in the BY and BY + EA groups; and the NF-*κ*B p65 activation was significantly decreased in the BY, EA, and BY + EA groups.

**Conclusions:**

Bufei Yishen granules and electroacupuncture have curative effects in COPD rats, and the combination therapy of Bufei Yishen granules and electroacupuncture is superior. The TLR-4/NF-*κ*B pathway may be involved in the potential mechanisms by which Bufei Yishen granules and electroacupuncture reduce inflammation.

## 1. Introduction

Chronic obstructive pulmonary disease (COPD) is a common, preventable, and treatable disease characterized by persistent respiratory symptoms and airflow limitation [1]. Due to its high morbidity and mortality, COPD has become a serious health problem globally. Approximately 65 million patients suffer from moderate to severe COPD worldwide [2]. A recent study in China showed that the overall prevalence of COPD is 8.6% and that the prevalence in people aged over 40 years old is as high as 13.7% [3]. In 2017, 3.91 million all-age deaths worldwide were due to chronic respiratory diseases, including 3.19 million deaths from COPD [4]. In addition, COPD seriously affects patients' quality of life and is one of the leading causes of disability adjusted life years (DALYs) [5]. Inflammation is central in COPD development and the release of inflammatory mediators and destructive enzymes by inflammatory cells implicated in the progressive destruction of the lung in COPD [6]. Toll-like receptors (TLRs) recognize different pathogen-associated molecular patterns and are involved in the initiation of innate and adaptive immune responses [7]. Several TLRs, including Toll-like receptor (TLR)-2, TLR-4, and TLR-9, participate in the pathogenesis of COPD, and particularly, TLR-4 is regarded as a major TLR responsible for sustaining the inflammatory responses in COPD [8, 9]. TLR-4 is activated in cigarette smoke-induced COPD, resulting in signal transduction cascades. Nuclear factor kappa B (NF-*κ*B) is the most important downstream pathway component that regulates the activity of cytokines, including interleukin (IL)-1*β*, IL-6, and tumor necrosis factor (TNF)-*α*, in airway pathology [10, 11]. TLR4/NF-*κ*B signaling plays an important role in inflammatory responses [12, 13]. Recently, a study demonstrated that airway inflammation in ovalbumin-induced mice was ameliorated through the inhibition of TLR4/NF-*κ*B signaling [14]. The TLR-4/NF-*κ*B pathway has been suggested to be a key target in the production and progression of airway inflammation.

The recommended pharmacotherapy for the treatment of COPD is generally bronchodilators (*β*2 agonists and long-acting anticholinergic agents) in patients with mild disease [15, 16]. Corticosteroids, antibiotics, bronchodilators, and oxygen therapy can benefit acute exacerbations of COPD, and inhaled corticosteroids (ICS) could be useful for COPD-asthma overlap syndrome patients [17–20]. Although the existing therapies can effectively improve symptoms, other than smoking cessation, to date, no treatment can suppress disease progression [21]. Traditional Chinese medicine (TCM), including Chinese medicine, acupuncture, acupoint-sticking, and pulmonary rehabilitation, represents important complementary and alternative medicine therapies for COPD that show potential advantages in improving symptoms, reducing the frequency of acute exacerbations, and improving exercise endurance and health-related quality of life [22–27].

Bufei Yishen granules (BY) comprise Chinese herbs that have been highly demonstrated to ameliorate lung and kidney functions, nourish Qi, and activate blood, resolve stasis and dissolve sputum. A four-center, open-label, randomized, controlled clinical study demonstrated that Bufei Yishen granules had evident and safe effects in COPD patients throughout the 12-month follow-up duration [28, 29]. Previous research has confirmed that such granules could also improve COPD patient self-efficacy and satisfaction [30]. The dramatic and long-term effects of BY on decreasing inflammatory responses, reducing pulmonary pathological impairment, and airway remodeling have been shown in a COPD rat model [31–34].

Acupuncture has been used as a clinical treatment for COPD for thousands of years [35]. Based on the “meridian theory” described in the Emperor's Classic of Internal Medicine, acupuncture could regulate Qi and dredge meridians to treat various diseases [36]. Modern life science technology has verified the biological values of acupuncture, including influencing gene expression, protein-protein interactions, and other biological processes [37, 38]. Electroacupuncture (EA) is a modern therapy involving acupuncture that could enhance efficacy by applying electrical stimulation to acupuncture points. In the treatment of COPD, acupuncture showed clinical effects in improving the exercise capacity and respiratory function and reducing dyspnea during exercise, especially in severe patients [39, 40]. Animal experiments have suggested that acupuncture could contribute to lung protection by regulating inflammatory cytokines in a smoke-induced COPD rat model [35, 41].

Internal-external combined therapy is well accepted and wildly used in TCM treatment for COPD. A 4-center, double-blinded, and randomized study showed that Bufei Yishen granules combined with acupoint sticking therapy had beneficial effects in COPD patients by ameliorating clinical symptoms, reducing acute exacerbation, and improving lung function and the dyspnea grade [42]. In long-term clinical practice, the combined therapy of Bufei Yishen granules and electroacupuncture shows beneficial and safe effects on COPD patients and is more advantageous in improving the clinical symptoms. This study aimed to investigate the effects of the combination of Bufei Yishen granules plus electroacupuncture in COPD rats based on the TCM internal-external combined therapy theory and determine the potential mechanisms via TLR-4/ NF-*κ*B signaling. This study's results may elucidate the effects and possible mechanisms of the combined therapy on COPD and provide evidence for COPD clinical treatment.

## 2. Materials and Methods

### 2.1. Animals

Forty-two male and forty-two female specific pathogen-free Sprague Dawley rats (weight: 250 ± 20 g; age: 3 months; animal permit number: 41003100004024) were purchased from the Laboratory Animal Center of Henan Province (SCXK [Henan] 2015–0004). The rats were housed in individual ventilated cages (Fengshi, Suzhou, China) for 7 days to adapt to the environment before the experimentation with free access to sterile food and water. The room was maintained at 25 ± 1°C with a relative humidity of 50 ± 10% and gas change at 10–15 times/hour. The experimental protocol was approved by the Experimental Animal Care and Ethics Committees of the First Affiliated Hospital, Henan University of Traditional Chinese Medicine, Zhengzhou, China.

### 2.2. Bacteria and Cigarettes

*Klebsiella pneumoniae *(strain ID: 46114) was purchased from the National Center for Medical Culture Collection (Beijing, China). The bacteria were cultured and prepared at a suspension of 6 × 10^8^ colony forming units (CFU) per milliliter with normal saline before administration to the rats [43].

Hongqiqu® filter cigarettes, containing 11 mg of tar, 0.9 mg of nicotine, and 11 mg of carbon monoxide, were purchased from Henan Tobacco Industry (Zhengzhou, China).

### 2.3. Drugs and Instrument

The Bufei Yishen granules were prepared and provided by the Pharmaceutical Department of Henan University of Chinese Medicine, Zhengzhou, China. The main compositions of the granules included Renshen (*Ginseng Radix et Rhizoma*; 9 g), Huangqi (*Astragali Radix*; 15 g), Yinyanghuo (*Epimedii Folium*; 9 g), Zhebeimu (*Fritillariae Thunbergii Bulbus*; 9 g), Chenpi (*Citri Reticulatae*; 9 g), and Chishao (*Paeoniae Radix Rubra*; 9 g). The herbs were extracted according to the standard operation procedure and then made into dry extract. Each 1 g dry extract contained 3.14 g of raw herbs. Additionally, aminophylline tablets (Xinhua, Shandong, China; 0.1 g/tablet), acupuncture needles (HUANQIU, Suzhou, China; size: 0.30*∗*13 mm), and an electroacupuncture apparatus (Hwato, Suzhou, China; type: SDZ-V) were used.

### 2.4. COPD Model Preparation and Administrations

Eighty-four Sprague-Dawley rats were randomly divided into seven groups, namely, the normal, model, aminophylline (APL), Bufei Yishen granules (BY), electroacupuncture (EA), Bufei Yishen granules + electroacupuncture (BY + EA), and sham acupuncture (SA) groups, with equal numbers of males and females per group.

The model, APL, BY, EA, BY + EA, and SA group rats were prepared to generate a COPD model by repeated cigarette smoke and bacterial exposure [43]. The rats were exposed to tobacco smoke at a smoke density of 3000 ± 500* ppm* for 30 min twice a day from week 1 to week 12.* Klebsiella pneumoniae* solution (0.1 mL, 6 × 10^8^ CFU/mL) was slowly dropped into the rat nostrils alternately every 5 days during the first 8 weeks. Meanwhile, the normal rats were exposed to fresh air and received 0.1 mL saline solution every 5 days.

From week 13 to week 20, the normal and model groups received 2 mL of 0.9% intragastric saline solution twice daily 6 days/week. The BY and APL groups were treated intragastrically with Bufei Yishen granules (3.7 g/kg/d, bid, 6 days/week) and aminophylline suspension (54 mg/kg/d, bid, 6 days/week), respectively. The EA and BY + EA groups underwent electroacupuncture treatment twice a week (on Monday and Thursday), while the BY + EA group also received Bufei Yishen granules (3.7 g/kg/d, bid, 6 days/week). The dosage of the Bufei Yishen granules and aminophylline was recalculated according to the weight on Monday, and the equivalent doses were calculated using the following formula: D_rat_ = D_human_ × (K_rat_/K_human_) × (W_rat_/W_human_)^2/3^, where D is the dose, K is the body shape index, and W is the weight.

**Figure 1:**
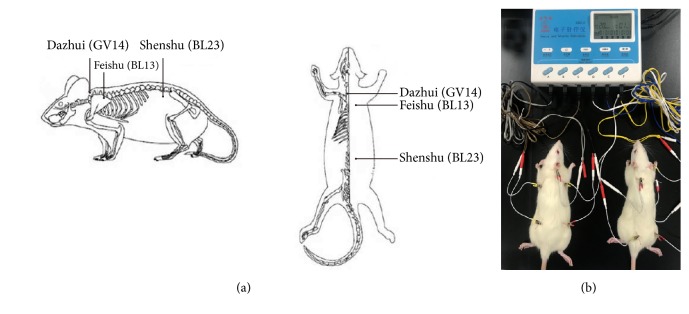
(a) Rat acupoint diagram. (b) Electroacupuncture operation diagram.

The electroacupuncture treatment was carried out as follows: the EA, BY + EA, and SA rat groups were mildly anaesthetized abdominally with 10% chloral hydrate at 2 mL/kg to ensure that all rats completely recovered from anesthesia within approximately 30 min [35]. Dazhui (GV 14, located on the posterior midline below the spinous process of the seventh cervical vertebra), Feishu (BL 13, located below and 3 mm lateral to the third thoracic vertebra on the back, bilateral), and Shenshu (BL 23, located below and 3 mm lateral to the second lumbar vertebra on the waist, bilateral) were selected as the acupoints [44] (Figure 1(a)). Stainless-steel needles were inserted to a depth of 4-5 mm at the acupoints and connected to an electroacupuncture apparatus with a 1-Hz alternating frequency and 1-mA intensity for 20 min (Figure 1(b)). The sham acupuncture group received the same grasping and same doses of the anesthetic but did not undergo EA.

### 2.5. Pulmonary Function

The tidal volume (VT), minute volume (MV), and peak expiratory flow (PEF) were detected every fourth week from week 0 to week 20 with an unrestrained whole body plethysmograph system (Buxco, NY, USA). The functional residual capacity (FRC), forced vital capacity (FVC), and forced expiratory volume at 0.1 s (FEV 0.1) were measured on the final day of week 20 by a FinePointe™ pulmonary function test system (Buxco, NY, USA).

### 2.6. Lung Tissue Morphology

Lung tissues were sampled from the right lower lobe, cut into 3-millimeter-thick slices along the maximum diameter, and fixed in 4% paraformaldehyde for 72 hours. Then, the lung tissues were embedded in paraffin wax, cut into 4-*μ*m-thick sections, and stained by routine hematoxylin-eosin (HE) processing. Six sections per group were observed and evaluated by optical microscopy and a photographic system (Olympus, Tokyo, Japan). The mean linear intercept (MLI) and mean alveolar number (MAN) were counted to determine the average value of the alveolar cavity and density of the alveoli. The MLI and MAN were calculated as follows: six random field photos were taken under an optical microscope using a photographic system at a 200× magnification per sample. A cross (+) was drawn on each photo through the center to count the length of the cross (L) and number of alveolar septa (Ns) on the cross. Then, the area of the visual field (A) was measured, and the number of alveoli in each visual filed (Na) was counted. MLI (*μ*m) = L/Ns; MAN (/mm^2^) = Na/A [44].

### 2.7. Inflammatory Factors

The levels of IL-6 in the serum and IL-1*β* in the bronchoalveolar lavage fluid (BALF) were assayed by enzyme-linked immunosorbent assays (ELISAs) according to the instructions (BOSTER, Wuhan, China). Serum samples from abdominal aorta blood were exposed to room temperature for 2 h, followed by centrifugation at 1500 rpm for 15 min. BALF was prepared by injecting 3 mL of 4°C normal saline into the left bronchus for perfusion, followed by pumping back into the centrifuge tube. The operation was repeated 3 times, and then, serum and BALF were collected for the inflammatory factor detection.

The expression levels of IL-6 and IL-1*β* in the lung tissues were detected by immunohistochemistry. After conventional deparaffinization and blocking with 3% H_2_O_2_ for 10 min to eliminate endogenous peroxidase activity, the lung tissue sections were subjected to antigen repair and 5% BSA solution blocking, followed by incubation with polyclonal anti-IL-6 (1:500 dilution; San Ying Biotechnology, Wuhan, China) and anti-IL-1*β* (1:500 dilution; San Ying Biotechnology, Wuhan, China) antibodies overnight at 4°C. On the following day, the slices were washed with phosphate buffer solution (PBS), incubated with biotin-labeled goat anti-mouse/rabbit immunoglobulin G (IgG) and stained with DAB solution. In each section, six random fields were photographed under an optical microscope using a photographic system. The IL-6 and IL-1*β* integral optical densities (IODs) were measured by Image-Pro Plus 6.0 (IPP 6.0) software (Media Cybernetics, Maryland, USA).

### 2.8. Real-Time PCR and Western Blotting Analysis

The mRNA expression levels of TLR-4, I*κ*B, and NF-*κ*B in the lungs were analyzed by quantitative real-time PCR (qRT-PCR) (normal, model, APL, BY, EA, and BY + EA groups). The primers were designed and synthesized by Genscript Biotech Co. Ltd. (Nanjing, China) and are shown in Table 1. The total RNA was extracted using a total RNA extraction kit (Solarbio, Beijing, China) according to the manufacturer's instructions. Reverse transcription (RT) was performed using a Hiscript® II First-Strand cDNA Synthesis Kit (Vazyme, Nanjing, China). The reactions were performed using an Applied Biosystems 7500/7500 Fast Real-Time PCR System (AB, CA, USA). The initial enzyme activation step was performed at 95°C for 5 min, followed by 40 cycles of 95°C for 10 s and 60°C for 30 s. At the end of qRT-PCR, the melting curve range was set to 95°C for 15 s, 60°C for 60 s, and 95°C for 15 s.

The protein expression levels of TLR-4, I*κ*B alpha (I*κ*B*α*), phosphorylated I*κ*B alpha (p-I*κ*B*α*), NF-*κ*B p65, and phosphorylated NF-*κ*B p65 (p-NF-*κ*B p65) in the lungs were measured by Western blotting (normal, model, APL, BY, EA, and BY + EA groups). After the total protein collection, the protein concentrations were detected using a BCA protein assay kit (Solarbio, Beijing, China). Protein denaturalization was performed at 100°C for 10 min with 2% SDS, and then, 5% of 2-mercaptoethanol was added. Subsequently, 40*μ*g of protein was separated by 10% sodium dodecyl sulfate-polyacrylamide gel electrophoresis (SDS-PAGE) and was transferred to polyvinylidene difluoride (PVDF) membranes (Millipore, MA, USA). After blocking with 5% nonfat dry milk, the blotted membranes were incubated with TLR-4 (1:500 dilution; Elabscience, Wuhan, China), I*κ*B*α* (1:1000 dilution; Elabscience, Wuhan, China), p-I*κ*B*α* (1:500 dilution; Elabscience, Wuhan, China), NF-*κ*B p65 (1:1000 dilution; GeneTex, CA, USA), p-NF-*κ*B p65 (1:500 dilution; GeneTex, CA, USA), and GADPH (1:5000 dilution; Proteintech, Wuhan, China) antibodies. The signals were visualized using Super ECL Plus reagent (Solarbio, Beijing, China) and scanned and quantified by a Chemi DocTM MP System (Bio-Rad, CA, USA).

### 2.9. Measurement of NF-*κ*B Activity

The DNA binding activity of NF-*κ*B was measured with a sensitive, nonradioactive transcription factor ELISA kit, i.e., the TransAM NF-*κ*B p65 kit (Active Motif, CA, USA). Whole-cell extracts of 30 *μ*g of lung tissues were incubated for 1 h in a 96-well plate, and an oligonucleotide containing the NF-*κ*B consensus site (5′-GGGACTTTCC-3′) was immobilized. Then, the primary NF-*κ*B antibody (1:1000 dilution) was added, and the samples were incubated for 1 h; subsequently, the samples were incubated with the secondary antibody (1:1000 dilution) for 1 h at room temperature. After the colorimetric reaction, the absorbance at 450 nm was read, and the blank value was subtracted. For the competition assays, the extracts were incubated with 2 *μ*L wild-type oligonucleotide or a mutated oligonucleotide.

### 2.10. Statistical Analysis

The data were analyzed by IBM SPSS22.0 and GraphPad Prism 7.0 software and are expressed as the means ± standard error (SE). One-way analysis of variance (one-way ANOVA) was employed for the multiple comparisons. The interaction between the Bufei Yishen granules and electroacupuncture was analyzed by a two-way analysis of variance (two-way ANOVA). The significant level was set as* P* < 0.05.

## 3. Results

### 3.1. Pulmonary Function

**Figure 2:**
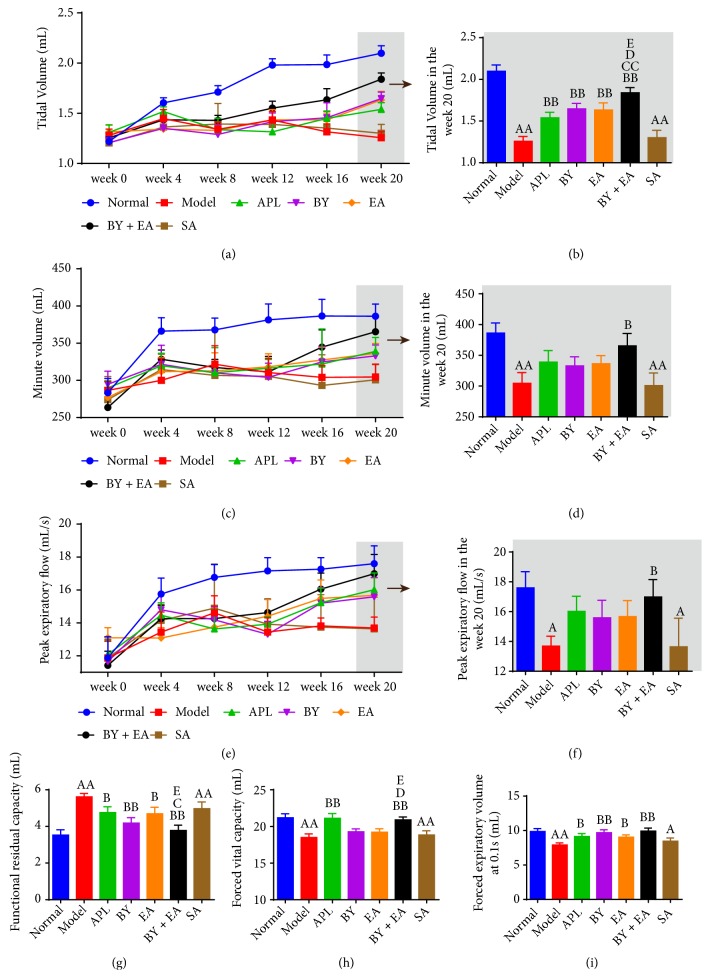
Changes in the pulmonary function in all groups. (a) Tidal volume (VT), (c) minute volume (MV), and (e) peak expiratory flow (PEF) in rats from week 0 to week 20. (b) Tidal volume (VT), (d) minute volume (MV), (f) peak expiratory flow (PEF), (g) functional residual capacity (FRC), (h) forced vital capacity (FVC), and (i) forced expiratory volume at 0.1 s (FEV 0.1) in rats at week 20. The data are expressed as the means ± SE (*n* = 8-12). ^A^*P* < 0.05 versus the normal group, ^AA^*P* < 0.01 versus the normal group; ^B^*P* < 0.05 versus the model group, ^BB^*P* < 0.01 versus the model group; ^C^*P* < 0.05 versus the APL group, ^CC^*P* < 0.01 versus the APL group, ^D^*P* < 0.05 versus the BY group, and ^E^*P* < 0.01 versus the EA group.

Decreased lung function is one of the most important clinical characteristics of patients with COPD. As shown in Figure 2, MV, VT, PEF, FVC, and FEV 0.1 in the model and SA groups were significantly lower than those in the normal group, but FRC was significantly increased (*P *< 0.05). After 8 weeks of treatment, VT, FRC, and FEV 0.1 in the treatment groups (APL, BY, EA, and BY + EA) showed improvement compared with those in the model group. MV and PEF were significantly increased in the BY + EA group, and FVC was improved in the APL and BY + EA groups (*P *< 0.05). VT in the BY + EA group was higher than that in the other treatment groups (*P *< 0.05). FRC in the BY + EA group was better than that in the APL and EA groups, and FVC in the BY + EA group was increased compared with that in the EA and BY groups (*P *< 0.05).

### 3.2. Lung Tissue Morphology

**Figure 3:**
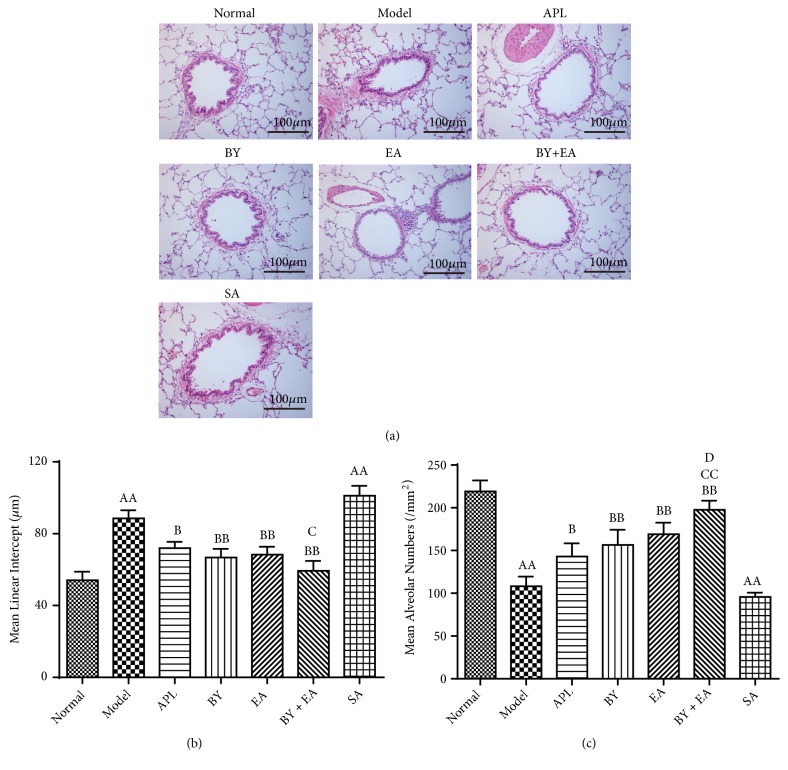
Changes in the lung tissue morphology in all groups. (a) Pathological changes in the lung tissues (HE, 200×). (b) Mean linear intercept (MLI). (c) Mean alveolar number (MAN). The values are expressed as the means ± SE (*n* = 6). ^AA^*P* < 0.01 versus the normal group; ^B^*P* < 0.05 versus the model group, ^BB^*P* < 0.01 versus the model group; ^C^*P* < 0.05 versus the APL group, ^CC^*P* < 0.01 versus the APL group, and ^D^*P* < 0.05 versus the BY group.

In this study, we observed lung tissue histopathological changes in the rats, which are shown in Figure 3(a). The structure of the bronchioles and alveoli in the normal group rats was complete, and few inflammatory cells were observed. The lung tissue in the model and SA groups showed obvious alveolar fracture and fusion, and the alveolar cavity was enlarged, which is a sign of moderate or severe emphysema. The treatment groups showed improvement with a complete alveolar structure and fewer inflammatory exudates than the model group.

As shown in Figures 3(b) and 3(c), compared with that in the normal group, the MLI in the model group was significantly enlarged, while the MAN was significantly decreased (*P *< 0.01). Compared with that in the model group, the MLI in the 4 treatment groups was significantly decreased, and the MAN in the 4 treatment groups was significantly increased (*P *< 0.05). Compared with that in the APL group, MLI in the BY + EA group was significantly decreased, and MAN was significantly increased (*P *< 0.05). MAN in the BY + EA group was higher than that in the BY group (*P *< 0.05).

### 3.3. Inflammatory Factors

**Figure 4:**
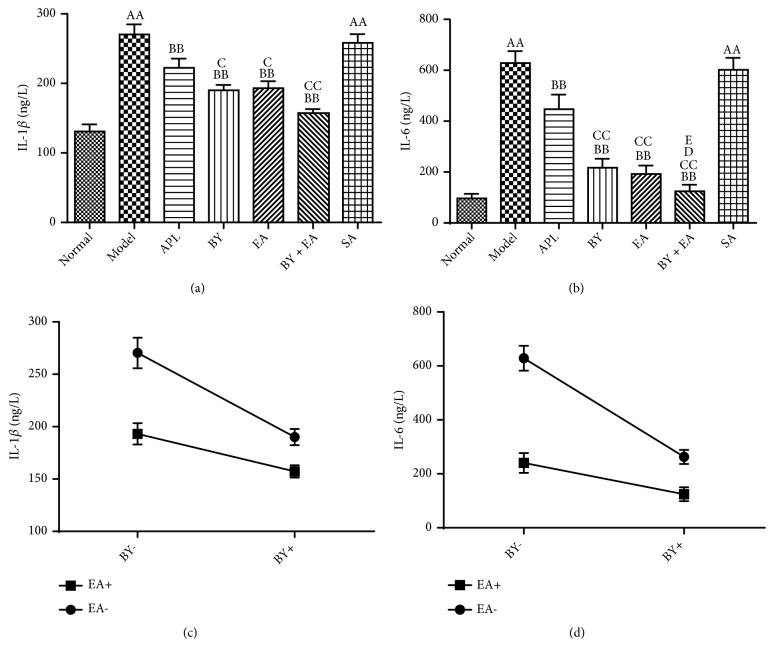
Changes in the inflammatory factor levels in all groups. (a), (b) Level of IL-1*β* in the BALF and IL-6 in the serum. (c), (d) Association between Bufei Yishen granules and electroacupuncture in regulating the IL-1*β* and IL-6 levels. Values are expressed as the means ± SE (*n* = 6-8). ^AA^*P* < 0.01 versus the normal group; ^B^*P* < 0.05 versus the model group, ^BB^*P* < 0.01 versus the model group; ^C^*P* < 0.05 versus the APL group, ^CC^*P* < 0.01 versus the APL group, ^D^*P* < 0.05 versus the BY group, and ^E^*P* < 0.01 versus the EA group.

COPD is characterized by chronic lung inflammation that results in progressive and irreversible airflow obstruction and exacerbations. As shown in Figures 4(a) and 4(b), the inflammatory factors IL-1*β* and IL-6 tested by ELISA in the model and SA groups were significantly higher than those in the normal group (*P *< 0.01). Compared with those in the model group, the levels of IL-1*β* and IL-6 in the treatment groups were significantly decreased (*P *< 0.05), while there was no difference between the SA and model groups. The levels of IL-1*β* and IL-6 in the BY, EA, and BY + EA groups were lower than those in the APL group (*P *< 0.05), and the level of IL-6 in the BY + EA group was significantly lower than that in the BY and EA groups (*P *< 0.05). The interaction between the Bufei Yishen granules and electroacupuncture in the regulation of the inflammatory factor levels is shown in Figures 4(c) and 4(d), and a significant decrease in the IL-1*β* and IL-6 levels was found with the Bufei Yishen granules plus electroacupuncture combination (*P *< 0.05).

**Figure 5:**
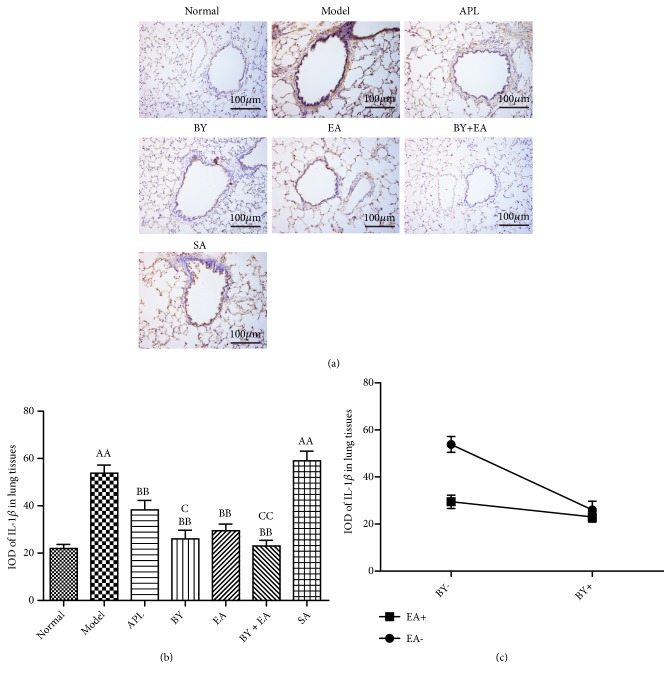
Expression levels of IL-1*β* in lung tissues. (a) Lung tissue immunohistochemistry photograph. Magnification at 200×. (b) Integral optical density (IOD) of IL-1*β*. (c) Association between Bufei Yishen granules and electroacupuncture in regulating the expression of IL-1*β* in lung tissues. The values are expressed as the means ± SE (*n* = 6). ^AA^*P* < 0.01 versus the normal group; ^B^*P* < 0.05 versus the model group, ^BB^*P* < 0.01 versus the model group; ^C^*P* < 0.05 versus the APL group, and ^CC^*P* < 0.01 versus the APL group.

**Figure 6:**
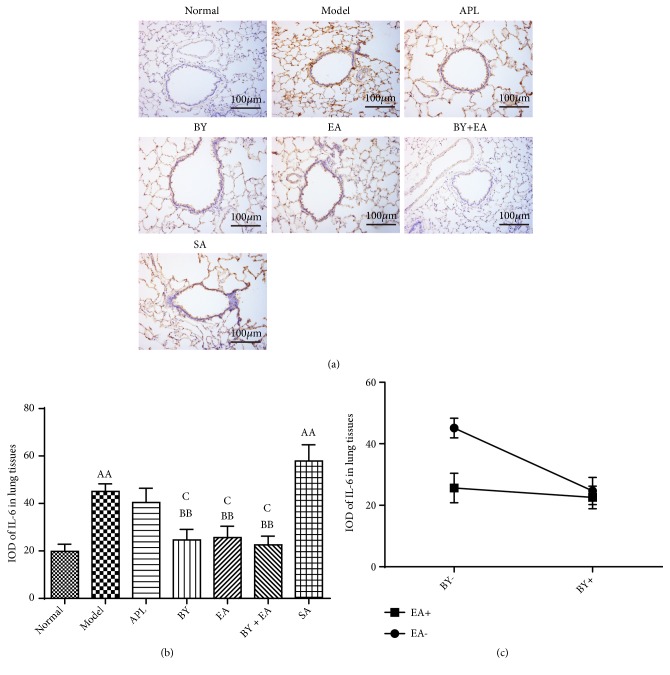
Expression levels of IL-6 in lung tissues. (a) Lung tissue immunohistochemistry photograph. Magnification at 200×. (b) Integral optical density (IOD) of IL-6. (c) Association between Bufei Yishen granules and electroacupuncture in regulating the expression of IL-6 in lung tissues. The values are expressed as the means ± SE (*n* = 6). ^AA^*P* < 0.01 versus the normal group; ^B^*P* < 0.05 versus the model group, ^BB^*P* < 0.01 versus the model group; ^C^*P* < 0.05 versus the APL group.

The expression levels of IL-1*β* and IL-6 in the lung tissues are shown in Figures 5 and 6. The IODs of IL-1*β* and IL-6 in the model and SA groups were significantly higher than those in the normal group (*P *< 0.01). Compared with those in the model group, the IODs of IL-1*β* in the APL, BY, EA, and BY + EA groups and IODs of IL-6 in the BY, EA, and BY + EA groups were significantly decreased (*P *< 0.01). The IODs of IL-1*β* in the BY and BY + EA groups were lower than those in the APL group (*P *< 0.05). The IODs of IL-6 in the BY, EA, and BY + EA groups were lower than those in the APL group (*P *< 0.05). As shown in Figures 5(c) and 6(c), an interaction was found between the Bufei Yishen granules and electroacupuncture in terms of decreased IL-1*β* and IL-6 expression (*P *< 0.05).

### 3.4. mRNA and Protein Expression Levels of TLR-4, I*κ*B, and NF-*κ*B in the Lung

**Figure 7:**
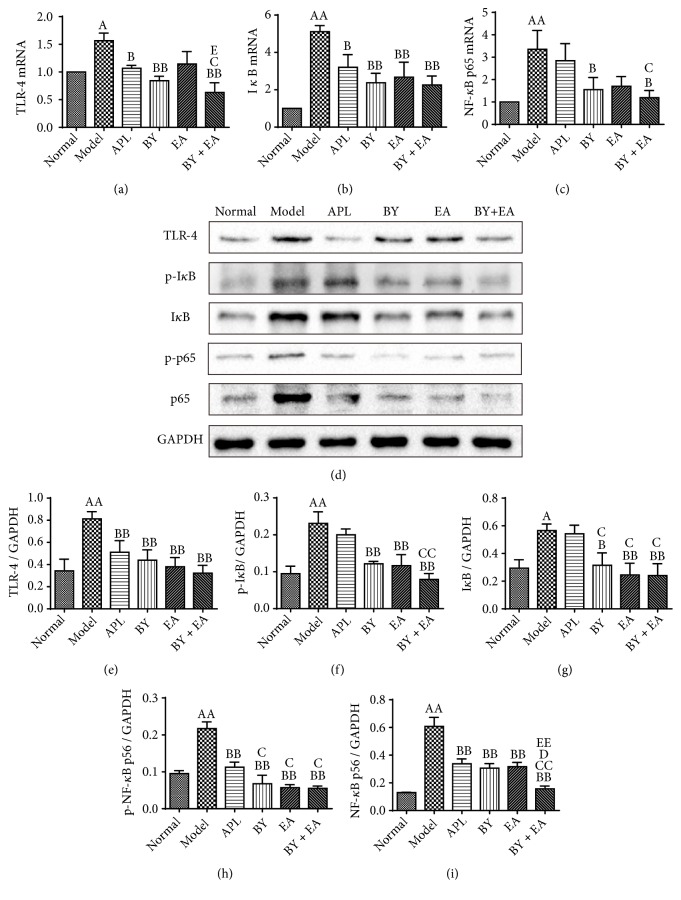
mRNA and protein expression levels of TLR-4, I*κ*B, and NF-*κ*B in the lung. (a)-(c) mRNA expression levels of TLR-4, I*κ*B, and NF-*κ*B (*n* = 6). (d)-(i) Protein expressions of TLR-4, I*κ*B*α*, p-I*κ*B*α*, NF-*κ*B p65, and p-NF-*κ*B p65 (*n* = 3). The values are expressed as the means ± SE. ^A^*P* < 0.05 versus the normal group, ^AA^*P* < 0.01 versus the normal group; ^B^*P* < 0.05 versus the model group, ^BB^*P* < 0.01 versus the model group; ^C^*P* < 0.05 versus the APL group, ^CC^*P* < 0.01 versus the APL group, ^D^*P* < 0.05 versus the BY group, and ^EE^*P* < 0.01 versus the EA group.

The TLR-4/NF-*κ*B signaling pathway has been proven to be related to the inflammation response. To further clarify the underlying mechanism of the association between the Bufei Yishen granules and electroacupuncture in COPD, activated proteins related to the TLR-4/NF-*κ*B signaling pathway were detected. As shown in Figures 7(a)–7(c), the mRNA expression levels of TLR-4, I*κ*B and NF-*κ*B in the model group were increased compared with those in the normal group (*P *< 0.05). Compared with those in the model group, the expression levels of TLR-4 and I*κ*B in the APL, BY, and BY + EA groups, and NF-*κ*B expression in the BY and BY + EA groups were decreased (*P *< 0.05). The mRNA expression levels of TLR-4 and NF-*κ*B in the BY + EA group were decreased compared with those in the APL group (*P *< 0.05). The mRNA expression of TLR-4 in the BY + EA group was decreased compared with that in the EA group (*P *< 0.05).

As shown in Figure 7(e), the protein expression of TLR-4 in the model group was higher than that in the normal group (*P *< 0.01). The protein expression of TLR-4 in the treatment groups (APL, BY, EA, and BY + EA) was significantly decreased (*P *< 0.01). As shown in Figures 7(f) and 7(g), the expression levels of I*κ*B*α* and p-I*κ*B*α* were increased in the model group and decreased in the BY, EA, and BY + EA groups (*P *< 0.05). The protein expression levels of p-I*κ*B*α* in the BY + EA group and I*κ*B*α* in the BY, EA, and BY + EA groups were lower than those in the APL group (*P *< 0.05). Compared with those in the normal group, the protein expression levels of NF-*κ*B p65 and p-NF-*κ*B p65 in the model group were significantly increased (*P *< 0.01). The expression levels of NF-*κ*B p65 and p-NF-*κ*B p65 in the treatment groups (APL, BY, EA, and BY + EA) were significantly decreased compared with those in the model group (*P *< 0.01). The expression level of NF-*κ*B p65 in the BY + EA group was lower than that in the BY, EA, and APL groups (*P *< 0.05) (Figures 7(h) and 7(i)).

### 3.5. NF-*κ*B DNA Binding Activity

**Figure 8:**
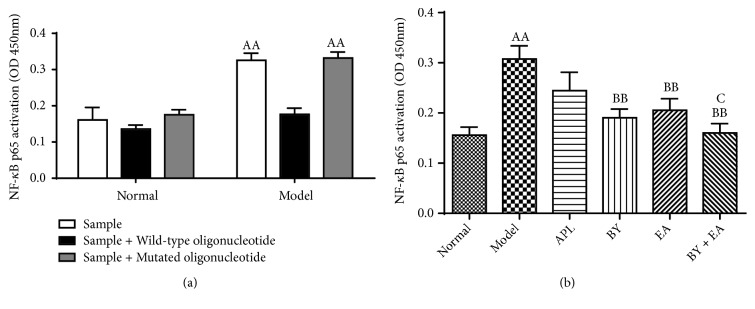
Changes of DNA binding activity of NF-*κ*B p65 in the lung. (a) The competition assay. The wild-type consensus oligonucleotide was added as a competitor for NF*κ*B binding in order to monitor the specificity of the assay, and the mutated consensus oligonucleotide had no effect on NF-*κ*B binding (*n* = 3). (b) Changes of DNA binding activity of NF-*κ*B p65 in the lung (*n* = 6). The values are expressed as the means ± SE. ^AA^*P* < 0.01 versus the normal group; ^BB^*P* < 0.01 versus the model group; ^C^*P* < 0.05 versus the APL group.

To further investigate the effect of BY and EA on NF-*κ*B signaling, the binding activity of the p65 subunit was also measured with a sensitive assay. As shown in Figure 8, the NF-*κ*B p65 activation was significantly increased in the COPD rats and prevented in the BY, EA, and BY + EA groups. The NF-*κ*B p65 activation in the BY + EA group was decreased than that in the APL group (*P *< 0.05) (Figure 8(b)). The competition assay showed that the DNA binding activity was prevented by wild-type consensus oligonucleotide, a competitor for NF*κ*B binding, and the mutated consensus oligonucleotide had no effect on NF*κ*B binding, which confirmed the specificity of the assay (Figure 8(a)).

## 4. Discussion

Currently, COPD remains a major public health problem. Regarding its treatment, traditional Chinese medicine, including internal treatment, such as Chinese herbal compounds, and external therapy, such as acupuncture, has garnered much attention [45, 46]. The purpose of this study was to explore the effect of acupuncture as an adjunct therapy combined with Chinese herbal compounds in the treatment of COPD model rats and to explore the possible underlying mechanism. BL 13 (Feishu), BL 23 (Shenshu), and GV 14 (Dazhui), which are the most common acupuncture points used in clinical practice, were selected as the treatment points in this study based on TCM theory [44, 47]. BL 13, BL 23, and GV 14 along with Bufei Yishen granules could improve shortness of breath and cough because of the nourishment of the lung and kidney in TCM theory.

The model was generated by cigarette smoke and bacterial exposure, and several features of COPD could be observed, including decreased pulmonary function, local emphysema associated with pulmonary and airway inflammation [43, 48]. However, due to some differences in respiratory physiology between humans and animals, and the heterogeneity in human presentations of the disease, rat models still have some limitations in replicating human diseases [49]. To verify the replication of the COPD model, we examined the rats' pulmonary function and lung tissue pathology. During the first four weeks, the lung function in the normal group significantly increased with age and weight, while that in the model group slowly increased. After 12 weeks of preparation, the pulmonary function in the model group rats was significantly decreased, suggesting irreversible airflow limitation in the COPD rats. After 8 weeks of treatment, the pulmonary function of the four treatment groups had improved, especially in the BY + EA group. The pathological findings were generally consistent with the pulmonary function results. The pathological results indicated that the rats in the model and SA groups developed more severe lung tissue injury, while the four treatment groups showed improvement, but the pathological lesions were not completely reversed.

Inflammation is a central feature of COPD that causes the activation and alteration in the airway and lung structural cells and the activation and recruitment of infiltrating inflammatory cells [50, 51]. Chronic airway inflammation is mainly characterized by neutrophil airway infiltrations, and IL-1*β* is a key mediator of neutrophilic airway inflammation in COPD by playing an important role in initiating and maintaining airway inflammation [52, 53]. The inflammatory cytokine IL-6 has been shown to be elevated in different lung diseases and is closely related to COPD [54–56]. The levels of IL-6 in BALF are reportedly significantly increased in patients with stable COPD compared with those in healthy control smokers [57]. The results of this study showed that the levels of IL-1*β* and IL-6 were much higher in the COPD rats, while the levels were significantly decreased in the BY, EA, and BY + EA groups, especially in the BY + EA group. The two-way ANOVA results suggested that Chinese medicine and acupuncture treatment are associated with decreasing levels of IL-1*β* and IL-6. These results suggest that BY, EA, and BY + EA could regulate the lung and systemic inflammation response in COPD rats and that the effect of the combination is better than that of Chinese medicine or electroacupuncture alone.

To further uncover the underlying mechanisms by which COPD inflammation was reduced, we focused on TLR-4/NF-*κ*B pathway-related molecules (because no difference was found in the efficacy between the SA and model groups, the SA group was not used for the mechanism studies). TLRs, including TLR-4, activate NF-*κ*B by recognizing pathogen-associated molecular patterns and damage-associated molecular patterns. Then, NF-*κ*B drives the inflammatory response by inducing the expression of proinflammatory and antiapoptotic genes [58]. Importantly, the increased epithelial expression of NF-*κ*B is associated with the severity of COPD, and during exacerbations of COPD, NF-*κ*B appears to be activated in patients' sputum macrophages [59, 60]. NF-*κ*B is a transcription factor of M1 macrophages and is required for the induction of numerous inflammatory genes, including TNF-*α*, IL-1*β*, IL-6, and IL-12 [61]. The regulation of the TLR-4/NF-*κ*B pathway may contribute to the inhibition of COPD inflammation. In this study, we found that the levels of TLR-4, p-I*κ*B*α*, and p-NF-*κ*B p65 were upregulated in the COPD rats and downregulated by the administration of BY combined with EA, and the combined therapy significantly reduced the NF-*κ*B DNA binding activity. It is likely that the anti-inflammatory effect of the BY and EA combination may be due to its regulation of the TLR-4/NF-*κ*B pathway, but other mechanisms may exist, requiring further investigation.

## 5. Conclusions

Therapy using Bufei Yishen granules, acupuncture, or a combination of Bufei Yishen granules and electroacupuncture demonstrates curative effects in COPD rats by improving lung function and pathology and reducing inflammatory responses. By regulating inflammation and improving the tidal volume and forced vital capacity, the effect of the combination is superior to that of Bufei Yishen granules or electroacupuncture alone. The TLR-4/NF-*κ*B pathway may be involved in the anti-inflammatory mechanism of BY, EA, and their combination. Other mechanisms underlying the combined therapy in COPD rats need further exploration.

## Figures and Tables

**Table 1:** Primer sequences of TLR-4, I*κ*B, NF-*κ*B, and GAPDH mRNA.

Gene	Primer	Primer sequence	Length
TLR-4	Forward primer	ACTCCATTCAAGCCCAAGCC	137
	Reverse primer	TCCCAAGATCAACCGATGGAC	
I*κ*B	Forward primer	CCAACCCAGGGAACGAAGAG	82
	Reverse primer	GGGGTGTGGCCATCATAGTT	
NF-*κ*B	Forward primer	TCATGCCCAACTTCTCCGAC	162
	Reverse primer	ATGCAATCCCACCGTAAGCA	
GAPDH	Forward primer	ACAGCAACAGGGTGGTGGAC	253
	Reverse primer	TTTGAGGGTGCAGCGAACTT	

## Data Availability

The data used to support the findings of this study are available from the corresponding author upon request.
